# Motto: Representing Motifs in Consensus Sequences with Minimum Information Loss

**DOI:** 10.1534/genetics.120.303597

**Published:** 2020-08-19

**Authors:** Mengchi Wang, David Wang, Kai Zhang, Vu Ngo, Shicai Fan, Wei Wang

**Affiliations:** * Bioinformatics and Systems Biology, University of California at San Diego, La Jolla, California 92093; † Department of Chemistry and Biochemistry, University of California at San Diego, La Jolla, California 92093; ‡ School of Automation Engineering, University of Electronic Science and Technology of China, Chengdu, China 610054; § Department of Cellular and Molecular Medicine, University of California at San Diego, La Jolla, California 92093

**Keywords:** consensus, information theory, motif, sequence logo, transcription factor binding

## Abstract

Sequence analysis frequently requires intuitive understanding and convenient representation of motifs. Typically, motifs are represented as position weight matrices (PWMs) and visualized using sequence logos. However, in many scenarios, in order to interpret the motif information or search for motif matches, it is compact and sufficient to represent motifs by wildcard-style consensus sequences (such as [GC][AT]GATAAG[GAC]). Based on mutual information theory and Jensen-Shannon divergence, we propose a mathematical framework to minimize the information loss in converting PWMs to consensus sequences. We name this representation as sequence Motto and have implemented an efficient algorithm with flexible options for converting motif PWMs into Motto from nucleotides, amino acids, and customized characters. We show that this representation provides a simple and efficient way to identify the binding sites of 1156 common transcription factors (TFs) in the human genome. The effectiveness of the method was benchmarked by comparing sequence matches found by Motto with PWM scanning results found by FIMO. On average, our method achieves a 0.81 area under the precision-recall curve, significantly (*P*-value < 0.01) outperforming all existing methods, including maximal positional weight, Cavener’s method, and minimal mean square error. We believe this representation provides a distilled summary of a motif, as well as the statistical justification.

MOTIF analysis is crucial for uncovering sequence patterns, such as transcription factor (TF) binding sites (Thompson *et al.* 2003), splicing sites (Murray *et al.* 2008), DNA methylation patterns (Wang *et al.* 2019), and histone modifications (Ngo *et al.* 2019b). A motif is typically represented as a position weight matrix (PWM), in which each entry shows the occurrence frequency of a certain type of nucleic acid at each position of the motif. PWMs are often visualized by the sequence logo (Schneider and Stephens 1990), which requires a graphical interface. However, when in a textual interface, representing PWMs requires an n by k matrix, where n is the number of characters (such as A, C, G, T, for nucleotides), and k is the length of the motif. Recently, several studies have shown the usefulness of representing motifs using kmers (Fletez-Brant *et al.* 2013; Ghandi *et al.* 2014; Zeng *et al.* 2016; Guo *et al.* 2018); despite the power of this representation in machine learning models, it is cumbersome to have a set of kmers to characterize a single motif. In many scenarios, motifs can be sufficiently represented by regular expressions of the consensus sequences, such as [GC][AT]GATAAG[GAC] for the GATA2 motif. This representation is the most compact and intuitive way to delineate a motif. In the GATA2 motif example, the GATAAG consensus in the center is the most prominent pattern that would be read off the PWM or sequence logo. For this reason, consensus sequences are still widely used by the scientific community. Consensus sequences in regular expression form are the only supported textual format to highlight motif occurrence in popular genome browsers such as UCSC (Kent *et al.* 2002) and IGV (Robinson *et al.* 2011). Consensus sequences are assigned to *de novo* motifs and sequences for informative denotations (Bailey *et al.* 2009; Heinz *et al.* 2010; Whitaker *et al.* 2015; Wang *et al.* 2019). Wildcard-like sequence patterns are also supported in DNA oligo libraries synthesis by major vendors including Invitrogen, Sigma-Aldrich, and Thermo-Fisher.

However, current methods that convert PWMs to consensus sequences are often heuristic. One simple approach is taking the nucleotide with maximal frequency at each position to define the consensus sequence (*e.g.*, GGTCAAGGTCAC for ESRRB). Unsurprisingly, this could misrepresent positions with similar frequencies (*e.g.*, 0.26, 0.25, 0.25, 0.24, which should have been assigned as N). Alternatively, in 1987, Cavener proposed to follow a set of rules: use the single nucleotide with the highest frequency when it exceeds 0.50 and two times the second-highest frequency; else, use the top two dinucleotides when their total frequencies exceed 0.75; else, use N (Cavener 1987). However, these rules are arbitrary, inflexible, and lack a mathematical framework.

Here, we present Motto, a sequence consensus representation of motifs based on information theory, which ensures minimal information loss when converted from a PWM (Figure 1). We provide a standardized solution that determines the optimal motif consensus sequence. We have also implemented a lightweight and easy-to-use Python package with versatile options for biologists.

**Figure 1:**
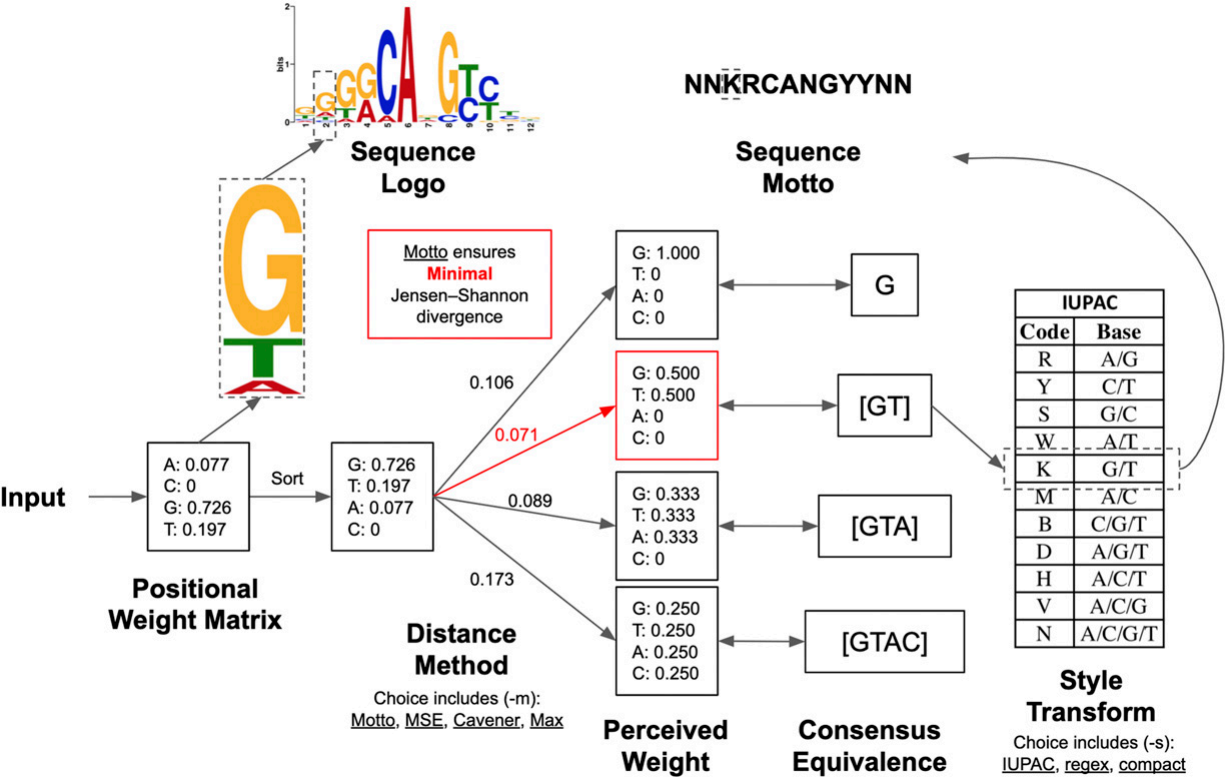
Overview of sequence Motto and comparison with sequence logo. Given a motif PWM as the input, Motto outputs a consensus that minimizes information loss. Here we show how the sequence Motto of the human transcription factor P73 is determined.

## Materials and Methods

A positional weight matrix (PWM) defines P(i,j), the probability of the jth character (out of n characters) at the ith position (out of k positions), where $\sum_{j}P\left(i,j\right)=1,$ i∈[1,k], and j∈[1,n]. For a given position i, let $M\left(i\right)=\left\{S_{i,1},\;S_{i,2},\;...,\;S_{i,m}\right\}$ denote a output consensus character set, where m is the number of characters to be presented, m∈[1,n], and let C(i, m) denote the perceived frequencies for a combination of m characters, defined by equal frequencies shared among included characters:

C(i,1)=C(i,2)=... =C(i,m) =1/m

For example, a M(i)={″A″,″C″, ″T″} is a case for m=3 and C(i, m)=0.333 with frequencies of [0.333, 0.333, 0, 0.333] for [A, C, G, T], respectively. Thus, we consider the optimal consensus sequence as a series of combination of characters M(i) that has the most similarity between C(i, m) and P(i) for each position i∈[1,k].

For convenience, in the following discussion we will omit the index i when possible, as we note that optimal M(i) is independent of the position i∈I under consideration. To further simplify the discussion, we use the second (i=2) position of the human TF P73 (Figure 1), where the P(″A″)=0.077, P(″C″)=0, P(″G″)=0.726, and P(″T″)=0.197, for demonstration below.

### Motto method using minimal Jensen-Shannon divergence

Here, we propose to use Jensen-Shannon divergence (JSD) to measure the similarity between C(m) and P. JSD has been widely used in information theory to characterize the difference between distributions (Lin 1991). Using this metric, the combination of nucleotides with the least JSD from C(m) to P will have the minimal “information loss,” and is thus considered as the optimal consensus nucleotide.

To efficiently compare JSD between all possible nucleotide combinations, we propose the following algorithm (Figure 1). First, we sort the nucleotides of the PWM in descending order, so that:

$$P\left(j_{1}\right)\geq P\left(j_{2}\right)\;...\;\geq P\left(j_{n}\right).$$

For example, at the second position of the human TF P73 (Figure 1), the nucleotides are sorted by occurrence frequencies so that:

P(″G″)=0.726 ≥P(″T″)=0.197 ≥P(″A″)=0.077≥P(″C″)=0.

Next, we reasoned that if a nucleotide with probability $P\left(S_{j}\right)$ is included in the output consensus sequence set, then all nucleotides with frequencies larger than $P\left(S_{j}\right)$ must also be included. Therefore, the optimal consensus character set M (denoted as $M^{\text{*}}$) is given by the optimal m(denoted as $m^{\text{*}}$), where:

$$M^{\text{*}}=\left(S_{1},\;S_{2},\;...,\;S_{m^{\text{*}}}\right)$$

$$P\left(S_{1}\right)\geq P\left(S_{2}\right)\;...\;\geq P\left(S_{m^{\text{*}}}\right).$$

For example, if $m^{\text{*}}=2$, then the optimal output character set will be $M^{\text{*}}=\;\left\{\prime \prime G\prime \prime ,\;\prime \prime T\prime \prime \right\},$ where P(″G″)=0.726 ≥P(″T″)=0.197.

The closer this distribution is to the original distribution of nucleotide frequencies, the better approximation of the consensus motif is to the original PWM. Thus, $m^{\text{*}}$ can be determined by minimizing the JSD between the two distributions:

$$m^{\text{*}}=argmin_{m}\left(JSD\left(C\left(m\right),\;P\right)+\;q^{2}\cdot m\right)$$

$$JSD\left(A,\;B\right)=\frac{1}{2}KLD\left(A,\;M\right)+\;\frac{1}{2}KLD\left(B,\;M\right)$$

$$M=\frac{1}{2}\left(A\;+\;B\right)$$

$$KLD\left(A,\;B\right)=\sum_{j=1}^{n}ln\left(\frac{A\left(j\right)}{B\left(j\right)}\right).$$

Here, q∈[0,1] is the ambiguity penalty, a parameter input from the user to penalize a larger m, in case a more definite output is preferred. When q=0(the default value), the optimal $m^{\text{*}}$ marks the canonical minimal JSD, which we deem to have retained the most information about the original PWM. When q=1, $m^{\text{*}}$ is guaranteed to be 1, thus the output consensus nucleotide is $M^{\text{*}}=\left\{S_{1}\right\},$ equivalent to using nucleotides with the maximal frequency.

Thus, the optimal consensus nucleotide set at the ith position is:

$$M^{\text{*}}=\left\{S_{1},\;S_{2},\;...,\;S_{m^{\text{*}}}\right\}.$$

Repeat this procedure for every position i∈[1,k], the final optimal consensus sequence is given by:

$$\left\{S_{1,\;1},\;S_{1,\;2},\;...,\;S_{1,\;m^{\text{*}}}\right\}\text{, }\left\{S_{2,\;1},\;S_{2,\;2},\;...,\;S_{2,\;m^{\text{*}}}\right\},...\text{, }\left\{S_{k,\;1},\;S_{k,\;2},\;...,\;S_{k,\;m^{\text{*}}}\right\}.$$

### Minimal mean squared error method

For comparison purposes, we have also implemented a minimal mean squared error (MSE) method, which is another metric used widely to measure distribution discrepancy (Lele 1993). The rest of the implementation is unchanged, except that the optimal m $\left(m^{\text{*}}\right)$ is now determined by minimizing the MSE between the two distributions:

$$m^{\text{*}}=argmin_{m}\left(MSE\left(C\left(m\right),\;P\right)+\;q^{2}\cdot m\right)$$

$$MSE\left(A,\;B\right)=\frac{1}{n}\sum_{j=1}^{n}{\left(A\left(j\right)-B\left(j\right)\right)}^{2}.$$

### Evaluating motif occurrence sites

We have collected 1156 common TFs from human and mouse from the databases of Transfac (Matys *et al.* 2006), Jaspar (Portales-Casamar *et al.* 2010), Uniprobe (Robasky and Bulyk 2011), hPDI (Xie *et al.* 2010), and HOCOMOCO (Kulakovskiy *et al.* 2018). Each PWM is converted into consensus sequences, using default options of the four discussed methods: JSD (described above), MSE (described above), Cavener (Cavener 1987), and the naive approach of using the maximal frequency. Motif occurrence sites are determined in the human genome (hg19), matched by their regular expressions. The ground truth of the occurrence sites is determined by scanning the original PWMs with FIMO (Grant *et al.* 2011) using a 1e-5 *P*-value cutoff. The resulting *P*-values are converted into a significance score [−log(*P*-value)] and assigned to the matched motif occurrence sites from sequence Mottos. Thus, the area under the precision-recall curves (Davis and Goadrich 2006) (auPRC) is calculated by comparing the motif occurrence sites and their significance scores. Resulting auPRCs are averaged and a paired (by each motif) *t*-test is conducted to determine performance. Comparisons with significance (*P*-value < 0.01) are shown (Figure 3).

### Data availability

The authors state that all data necessary for confirming the conclusions presented in the article are represented fully within the article. Motto is freely available at https://github.com/MichaelMW/motto. Motto representation of all 1156 common TFs, as well as their sequence logo used in this study can be found at http://wanglab.ucsd.edu/star/motto.

## Results and Discussion

Motto takes the MEME format of PWM as the input because of its popularity. The MEME format is supported by the majority of the motif databases (Kulakovskiy *et al.* 2018), and the MEME suite provides packages for integrative analysis and conversion from other motifs formats (Bailey *et al.* 2009). The recently proposed kmer-based motif models also support conversion to MEME format (Fletez-Brant *et al.* 2013; Ghandi *et al.* 2014; Zeng *et al.* 2016; Guo *et al.* 2018). Our package is lightweight and open-source. The algorithm is efficiently implemented in Python and the conversion for 1000 motif sequences typically takes <2 sec. In addition, perhaps expectedly, downstream analysis like matching motif occurrences using Motto is much faster (∼5 sec for a common PWM on a chromosome, implemented inhouse with Python) than a conventional PWM scanning (about 1 min, scanned with FIMO (Grant *et al.* 2011)). By default, the Motto package takes a motif in the MEME format, parses the header to get the nucleotide, computes the optimal consensus sequences based on the Motto method, and then outputs the sequence in a compact format (Figure 1). Motto provides flexibility at each step along this process. Input can be from a file, or from standard input, and Motto can consider nucleotides, amino acids, and customized characters such as CpG/non-CpG methylation (Ngo *et al.* 2019a) and protein phosphorylation (Amanchy *et al.* 2011). We have provided four methods for comparison: maximal probability (Max), heuristic Cavener’s method (hereafter referred to as Cavener), minimal MSE method, and our proposed Motto method using the minimal JSD (Motto) (see *Materials and Methods*). Three output styles are provided: (1) IUPAC uses a single character to represent the combination of nucleotides (*e.g.*, S for [CG]) and is the most compact form, but requires reference to the nomenclature (Johnson 2010); (2) regular expression (“regex”) enumerate all output consensus nucleotide ranked by occurrences and is recommended for downstream analysis, such as motif occurrence and oligo designs; (3) “compact” (the default) is the same as “regex,” except that it replaces [ACGT] with N. To trim off Ns ([ACGT]s) at both ends of the output sequences, a optional flag “–trim” is provided. If the users prefer consensus with more certainty (*e.g.*, prefer [AC] to [ACG]), they can use either “–maxCharacter” as a hard limit to the number of characters allowed, or use “–penalty” to penalize ambiguity (see *Materials and Methods*).

**Figure 2:**
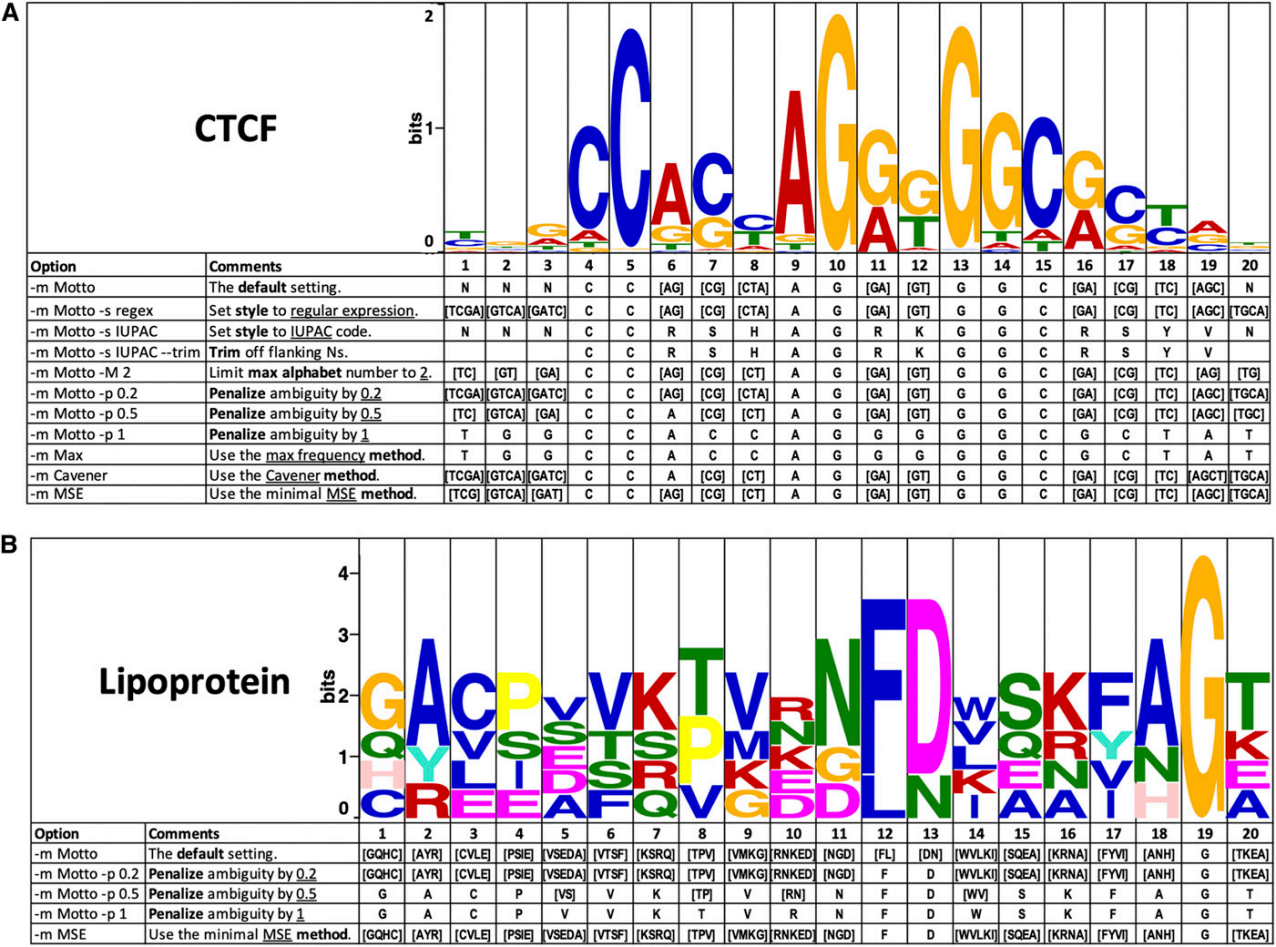
Example usage using human CTCF (upper panel) and lipoprotein binding sites from Bailey and Elkan (1994) (lower panel). The original PWM is shown in a sequence logo. Different Motto options resulted in various consensus sequence output at each position. In particular, “-m/–method” specifies the method: Motto (default), MSE (minimal mean square error), Cavener (Cavener 1987), or Max (using maximal frequency at each position); “-s/–style” specifies the output style: IUPAC (Johnson 2010) (single character for nucleotide combinations), regex (regular expression), or compact (convert [ACGT] to N in regex); “-t/–trim” is an option for trimming off the flanking Ns; “-p/–penalty” specifies a weight between 0 and 1 that penalizes ambiguity at each position (for details see *Materials and Methods*).

Effects of these options are shown using an example of human TF CTCF (Figure 2, upper panel). Unsurprisingly, MSE, Motto, and Cavener are more representative than the naive maximal probability methods. For example, positions 1, 2, 3 and 20 with low information content (<0.2) in CTCF, are justifiably called as “N” by Motto and Cavener, which is an improvement over strictly calling the top nucleotide. MSE considers [TCG] and [GAT] more representative at the first and the third position but agrees with Motto and Cavener at the 2nd and 20th. Similarly, Motto, MSE, and Cavener successfully capture strong double-consensus patterns at indices 7, 11, 12 and 16, which maximal probability fails to capture. The advantage of Motto over Cavener is noticeable at index 6, where the logo of CTCF shows a dominating AG consensus. While Motto finds this co-consensus, Cavener disregards G that barely misses the cutoff. In addition, at index 19, the logo of CTCF shows a strong three-way split among A, G and C, but Cavener, by its rules (as described previously), ignores all such triple patterns. In addition, among the four methods, only the Motto and MSE are capable of generating consensus sequences for amino acid motifs (Bailey and Elkan 1994) (Figure 2, lower panel). Due to its arbitrary nature, heuristic methods like Cavener have difficulties defining decision boundaries for motifs of more than four nucleotides or customized character sets found in motifs containing methylated DNA (Ngo *et al.* 2019a) and phosphorylated amino acids (Amanchy *et al.* 2011). In such cases, Motto and MSE provide more mathematically rigorous information than Cavener and oversimplified maximal consensus methods. With increased penalty level at 0, 0.2, 0.5,and 1 respectively, the consensus sequence smoothly progresses toward single nucleotide consensus (Figure 2). Such flexibility gives an advantage to users that are biased toward more defined consensus results.

**Figure 3:**
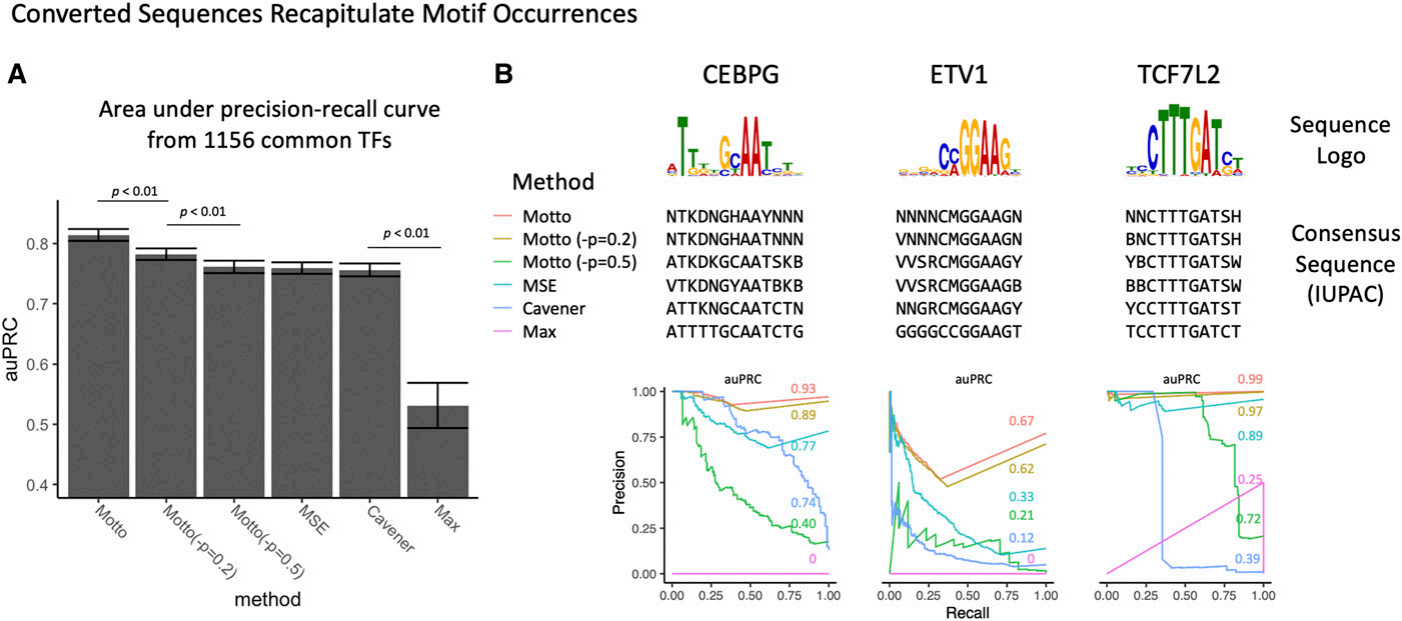
Converted sequence Mottos recapitulate motif occurrence sites of 1156 common human and mouse transcription factors (TFs) in the human genome (hg19). (A) The averaged area under the precision-recall curve (auPRC) using Motto (default method with minimal JSD, ambiguity penalty at -*P* = 0.2, and at -*P* = 0.5) compared with existing alternative methods. *P*-value determined by paired *t*-test. (B) Comparison in three examples TFs showing the differences of consensus sequences [shown in IUPAC (Johnson 2010) coding for better alignment] and performances.

To quantify how well these four methods summarize the information in the original PWMs, we converted 1156 common human and mouse TFs to consensus sequences and compared their matched occurrences (by regular expression) in the human genome (hg19) with conventional motif sites scanned by FIMO (Grant *et al.* 2011) with PWMs, which is how conventionally motif sites are determined (see *Materials and Methods*). We observe that using the Motto method has resulted in the best (0.81 ± 0.01) area under the Precision-Recall curve (auPRC), significantly (*P*-value < 0.01) better than existing alternative methods, including MSE (0.76 ± 0.01), Cavener (0.76 ± 0.01), and maximal frequency (0.53 ± 0.04) (Figure 3). In addition, we observed Motto performs better with lower ambiguity penalty, where the default setting with minimal ambiguity penalty (–*P* = 0) performs significantly (*P*-value < 0.01) better than setting penalty at 0.2 (0.78 ± 0.01) or 0.5 (0.76 ± 0.01) (Figure 3). This is consistent with the finding that setting the ambiguity penalty to the maximal value of 1 (–*P* = 1) is equivalent to using the max frequency method, resulting in the worst performance. These results confirm that Motto conversion minimizes information discrepancy (per JSD) from the original PWM, while setting larger penalties will result in more determined sequences at the cost of accurately recapitulating the original PWM.

In summary, Motto provides a mathematical framework and a set of convenient features to textualize PWMs in a compact, intuitive and accurate manner.
